# Photon irradiation prompts autophagy in anaplastic thyroid cancer

**DOI:** 10.1186/s12885-026-16762-0

**Published:** 2026-08-25

**Authors:** Sabine Wächter, Franziska Knauff, Silvia Roth, Norman Krasser-Gercke, Katrin Roth, Ali Ebrahimifard, Detlef K. Bartsch, Pietro Di Fazio

**Affiliations:** 1 Department of Visceral, Thoracic and Vascular Surgery, Philipps University Marburg, Baldingerstrasse, Marburg, 35043 Germany; 2 Core Facility Cellular Imaging, Center for Tumor Biology and Immunology, Philipps University Marburg, Hans-Meerwein Strasse 3, Marburg, 35043 Germany; 3 Department of Nuclear Medicine, Philipps University Marburg, Baldingerstrasse, Marburg, 35043 Germany

**Keywords:** Autophagy, Radiotherapy, Anaplastic thyroid cancer, Poor prognosis, Targeted therapy

## Abstract

Although promising results have been obtained for anaplastic thyroid cancer (ATC) therapy, this therapy still needs to be improved. In addition to the currently approved therapies, radiotherapy alone or combined with adjuvant immunotherapy could be beneficial for patients affected by ATC. The patients affected by this aggressive solid malignancy could benefit from the modulation of autophagy in cancer cells. This study focused on detecting autophagy players in ATCs and bursting autophagy process via photon irradiation to induce decay in irradiated ATCs. The transcript expression of autophagy genes was detected in tumor tissue resected from 19 patients and in C643 cells, four primary ATC cell lines and primary follicular thyroid cell line (Nthy-ori-3–1) photons irradiated with 4 or 6 Gy. The protein level of Beclin1 was detected by immunofluorescence in 10/19 patients. The levels of autophagy markers were detected by RT‒qPCR and western blotting in irradiated cells. Autophagy and maturation of autophagosome vesicles were monitored in C643 cells stably transfected with the GFP-RFP-LC3B plasmid. All patients included in the study exhibited significant overexpression of autophagy transcripts. Additionally, the Beclin1 protein was expressed in resected tumor tissue. Furthermore, the analysis of autophagy-related gene transcripts revealed significant increases in the expression of these genes in C643, Patient 2 and Patient 3 cells irradiated with 4 or 6 Gy. Additionally, irradiation with 4 or 6 Gy downregulated the expression of all the proteins involved in the autophagy process. Thus, the ongoing catabolic process was confirmed. Interestingly, the levels of AMPKα and its active phosphorylated form were strongly downregulated, which excluded its involvement in autophagy activation. 6 Gy photon irradiation caused an increase in both green and red fluorescence in C643-derived spheroids. An increase in fluorescence was detectable for up to 28 days. The spheroids gradually exhibited an increase in fluorescence, which probably caused the dismantling of their ultrastructure. This finding provides evidence of the bursting of autophagy and its ability to affect spheroid integrity. Photon irradiation exacerbates autophagy in anaplastic thyroid cancer cells and could represent a valid target for focused therapy against this aggressive malignancy.

**Supplementary Information:**

The online version contains supplementary material available at 10.1186/s12885-026-16762-0.

## Background

Although anaplastic thyroid cancer (ATC) accounts for only 1–2% of all thyroid cancers (TCs), it contributes to a large proportion of all patients who die from TC [1, 28]. The aggressive growth of this rare carcinoma is characterized by rapid lymphogenic and hematogenic metastasis with infiltration of surrounding structures,consequently, patients have a poor prognosis, with a median survival of only 3 to 5 months after initial diagnosis [10, 31].

In the last two decades, ATC therapy has been based on a variety of treatment options, including surgery, radiotherapy and chemotherapy. Photon radiotherapy is the standard treatment for ATC and is associated with prolonged overall survival at high doses (≥ 60 Gy). As a result, intensity-modulated radiotherapy (IMRT) has become increasingly important for the treatment of ATC, as it has been shown to provide high and precise radiation intensity to tumors while sparing surrounding healthy tissue from nonspecific side effects [1, 7, 11, 13, 26].

However, despite targeted, individualized treatment options, including IMRT, the poor prognosis of ATC has not significantly improved in the last 10 years [17].

In contrast, the development of targeted treatment options such as immune checkpoint inhibitors (ICIs) and multikinase inhibitors (mKIs) appears to improve survival in patients with advanced or primarily unresectable ATC, which has already been exhausted by canonical therapy options [20].

A previous study by our working group showed that photon radiation led to a decrease in cell viability in ATC cells alone or in combination with the ICI atezolizumab [30]. According to current knowledge, radiation induces DNA damage through double-strand breaks (DSBs) and activates the intrinsic and extrinsic apoptotic pathways [19].

In contrast, we were unable to detect any induction of apoptosis in irradiated ATCs [30]. Accordingly, other cellular processes must be involved in photon-induced cell death in ATC.

Therefore, this study focused on investigating whether photon irradiation can induce autophagy as a substitute mechanism for ATC cell death.

## Materials and methods

### Cell lines

C643 human anaplastic thyroid carcinoma cells kindly donated by Prof. A. Zielke (Diakonie-Klinikum Stuttgart; Stuttgart, Germany) and the human thyroid follicular epithelial cell line Nthy-ori-3–1 (MERCK—Sigma‒Aldrich Chemie GmbH, Schnelldorf Germany) were grown in RPMI 1640 (Gibco® by Life TechnologiesTM, Carlsbad, USA) supplemented with 10% fetal bovine serum (Gibco) and 10 U/ml penicillin and 100 µg/ml streptomycin (Gibco). The cells were kept under standard conditions (37 °C, 5% CO_2_) and routinely tested for Mycoplasma contamination [29, 30].

### Patient samples

Snap-frozen and formalin-fixed paraffin-embedded (FFPE) tumor tissue was collected from 19 patients affected by ATC who underwent surgical resection at the University Hospital Marburg between 2003 and 2023 [29].

### Preparation of patient-derived human tumor tissue (PDTT)

Patient-derived human tumor tissue (PDTT) was isolated from four surgically operated patients affected by ATC as previously described [32]. All PDTTs were obtained from patients who were never treated with neoadjuvant therapy. The tumor tissue was immediately collected in 50 ml falcon tube with sterile phosphate-buffered saline (PBS) without Ca^2+^ or Mg^+^ (L1825 Biochrom, Berlin, Germany). The tissue was washed 3 times with sterile PBS to remove any tissue debris or blood. Afterward, the tissue was cut into small pieces with a sterile scalpel (Feather, Osaka, Japan). The small pieces were rinsed through a cell strainer (352,350 BD Labware, Franklin Lakes, NJ, USA) and washed with Roswell Park Memorial Institute 1640 (RPMI1640) medium (FG1215 Biochrom, Berlin, Germany). The cell suspension was centrifuged at 1,500 rpm for 8 min at room temperature. The pellet was suspended in complete growth medium RPMI 1640 (Biochrom) supplemented with 10% fetal bovine serum (FBS,Biochrom), 10 U/mL penicillin and 100 g/mL streptomycin (Biochrom). The suspension was pipetted into a cell culture 6-well plate (83.3920 Sarstedt, Nümbrecht, Germany). After 2 h, the cell adhesion was monitored under contrast light microscope. Fresh medium was added regularly every second day. The cells were then trypsinized and transferred to 25 cm^2^ flasks and were grown in RPMI 1640 (Biochrom) supplemented with 10% fetal bovine serum (FBS; Biochrom), 10 U/mL penicillin and 100 g/mL streptomycin (Biochrom) under standard conditions (37 °C, 5% CO_2_). All cells were routinely tested for Mycoplasma contamination [32].

### Immunofluorescence staining of paraffin-embedded tissue

Two-micron sections of 4% formaldehyde-fixed paraffin-embedded tumor tissue were cut, rehydrated and deparaffinized. Antigen retrieval was performed in citrate buffer (pH = 6) in a microwave at 480 W for 10 min. Endogenous peroxidase activity was blocked with 3% H_2_O_2_ for 10 min. The sections were permeabilized with 0.5% Triton X-100 (Carl Roth Gmbh & Co. KG) in PBS (Life Technologies) for 10 min. Unspecific binding was blocked through a 30-min incubation in 10% immunized serum. The slides were then incubated with a 1 µg/ml primary antibody against Beclin1 (ab114071; Abcam, Cambridge UK) in 1% BSA-PBS-0.5% Tween 20 overnight at 4 °C. The bound primary antibody was labeled with 2 µg/ml Alexa Fluor® 488 goat anti-mouse IgG (H + L) secondary antibody. Nuclei were stained with 1 µg/ml Hoechst 33,342 (Sigma‒Aldrich) in 1% BSA-PBST. After 90 min of incubation with the secondary antibody and Hoechst, the tissue slides were processed with a Vector® TrueVIEW™ Autofluorescence Quenching Kit (VECTOR Laboratories, Burlingame, USA) and mounted with VECTASHIELD® Vibrance™ Antifade Mounting Medium (VECTOR Laboratories). LAS AF and LAS X software (Leica Microsystems, Wetzlar Germany) was used for the analysis of fluorescence images acquired with a wide-field fluorescence microscope (Leica DM 5500) [22].

### Irradiation

The cells were irradiated with an XRad 320iX irradiation cabinet (Precision X-ray, Inc., Denver, USA) at 8 mA and 320 kV at a dose rate of 1.0 Gy/min. A filter with 0.5 mm Al/0.5 mm Cu was employed [30].

### Quantitative RT‒PCR

Total RNA was isolated from the cells and 19 tumor tissue samples by using an RNeasy Mini Kit (74,106, QIAGEN, Hilden Germany) according to the manufacturer`s protocol. cDNA was reverse transcribed by using an iScriptTM cDNA Synthesis Kit (170–8891; Bio-Rad, Hercules, USA) on a FlexCycler (Analytik Jena AG, Jena, Deutschland). The primers used for human *BECN1* (QT00004221), *UVRAG* (QT00034328), *MAP1LC3B* (QT00055069), *SQSTM1* (QT00095676), *TFEB* (QT00069951), *PRKAA1_1* (QT00009436), *PRKAA2_1* (QT00042077) and *GAPDH* (QT01192646) were mixed with the GoTaq® qPCR Master Mix (Promega, Madison, USA) on an RT‒qPCR thermocycler CFX96TM Real-Time System (Bio-Rad Laboratories, Hercules, California USA). The results were analyzed with a Bio-Rad CFX-Manager (Bio-Rad Laboratories) and normalized to the GAPDH mRNA content for each sample. The raw data were further processed with Rest2009 (relative expression software tool V.2.0.13, Qiagen) [29].

### Western blot analysis

Whole-cell lysates were isolated in Jie´s Buffer (10 mM NaCl, 0.5% NonidetP40, 20 mM Tris–HCL (pH 7.4), 5 mM MgCl2, 1 mM PMSF, Complete Protease Inhibitor and Phosphatase Inhibitor (Roche, Basel Switzerland). The proteins were separated through SDS‒PAGE (NP0342, Life Technologies, Carlsbad, California, USA) and transferred to 0.2 µm nitrocellulose membranes (#1,704,158, Trans-Blot Turbo Transfer Pack, Bio-Rad Laboratories, USA) by semidry blotting with a Trans-Blot® TurboTM Transfer System (Bio-Rad Laboratories). The membranes were further sliced according to the molecular weight of the proteins of interest, blocked in 4% BSA (23,208; Thermo Fisher Scientific, Waltham, MA, USA) in TBS-Tween 20 (0.5%) and incubated with primary antibodies against Beclin1 (ab114071; Abcam), UVRAG (U7508. Sigma-Aldrich, St. Louis, USA), LC3B (ab51520, Abcam), SQSTM1 (ab96706, Abcam), AMPK-α (2532S, Cell Signaling Technology, Danvers, USA); phospho-AMPK-α (T172) (2525S; Cell Signaling Technology); and β-actin (A5441; Sigma‒Aldrich, St. Louis, USA). The bound primary antibodies were detected by secondary horseradish-labeled goat anti-rabbit (A0545, Sigma‒Aldrich) and goat anti-mouse (A9917, Sigma‒Aldrich) antibodies and SuperSignal West Pico Chemiluminescent Substrate (Thermo Fisher Scientific, Waltham, USA). The resulting bands were quantified by using Fusion image capture (VILBER LOURMAT Deutschland GmbH, Eberhardzell, Germany) and a Bio1D analysis system (VILBER LOURMAT Deutschland GmbH) [29].

### Stable transfection

C643 cells were stably transfected with an *E. coli* plasmid encoding RFP-GFP-MAP1LC3B (ptfLC3 was a gift from Tamotsu Yoshimori [Addgene plasmid #21,074; http://n2t.net/addgene:21074]; RRID:Addgene_21074) [12] by incubation with 20 µg/ml plasmid in serum-free medium and FUGENE® HD Transfection Reagent (Promega). Fresh medium containing the selective agent G-418 (Roche Diagnostics Gmbh, Risch-Rotkreuz, Switzerland) was added 96 h after transfection. After 1 week of G418-dependent selection, the transfected cells (fluorescent) were collected by scratching with a pipette under a fluorescence microscope. The scratched cells were plated on a new dish with fresh medium containing 20 µg/ml G-418 [6, 22].

### Autophagy assay

A total of 5,000 C643 cells stably transfected with RFP-GFP-MAP1LC3B were seeded in a round bottom low-attachment plate (Corning Spheroid Microplate 4515, Corning, USA) for 4 days. The transfected cells were firstly grown as spheroids and were then irradiated with 6 Gy. The green and red fluorescence intensity was continuously acquired by an IncuCyte® S3 Live-Cell Analysis System (Sartorius, Göttingen, Germany) and with a confocal microscope (Leica TCS SP8) by using a 488 nm laser (GFP) and 552 nm laser (RFP) with 5 × objective One planar in the middle of the spheroids [22].

### Protein interaction

Maps showing the protein interactions of the autophagy players were generated and downloaded from STRING v12.0.

### Statistical analysis

Unless otherwise stated, all the experiments were performed in triplicate and repeated at least three times. The data were collected using Excel (Microsoft Office). Significance was calculated using the 2-way ANOVA and Dunnett’s post hoc test for paired samples. *P* < 0.05 was considered to indicate statistical significance (*).

### Ethical approval

The study was conducted according to the guidelines of the Declaration of Helsinki and approved by the Institutional Ethics Committee of University Hospital of Marburg (No. 123/19). Informed consent was obtained from all subjects involved in the study.

## Results

### Expression of autophagy players in ATC patient tissue

**Fig. 1:**
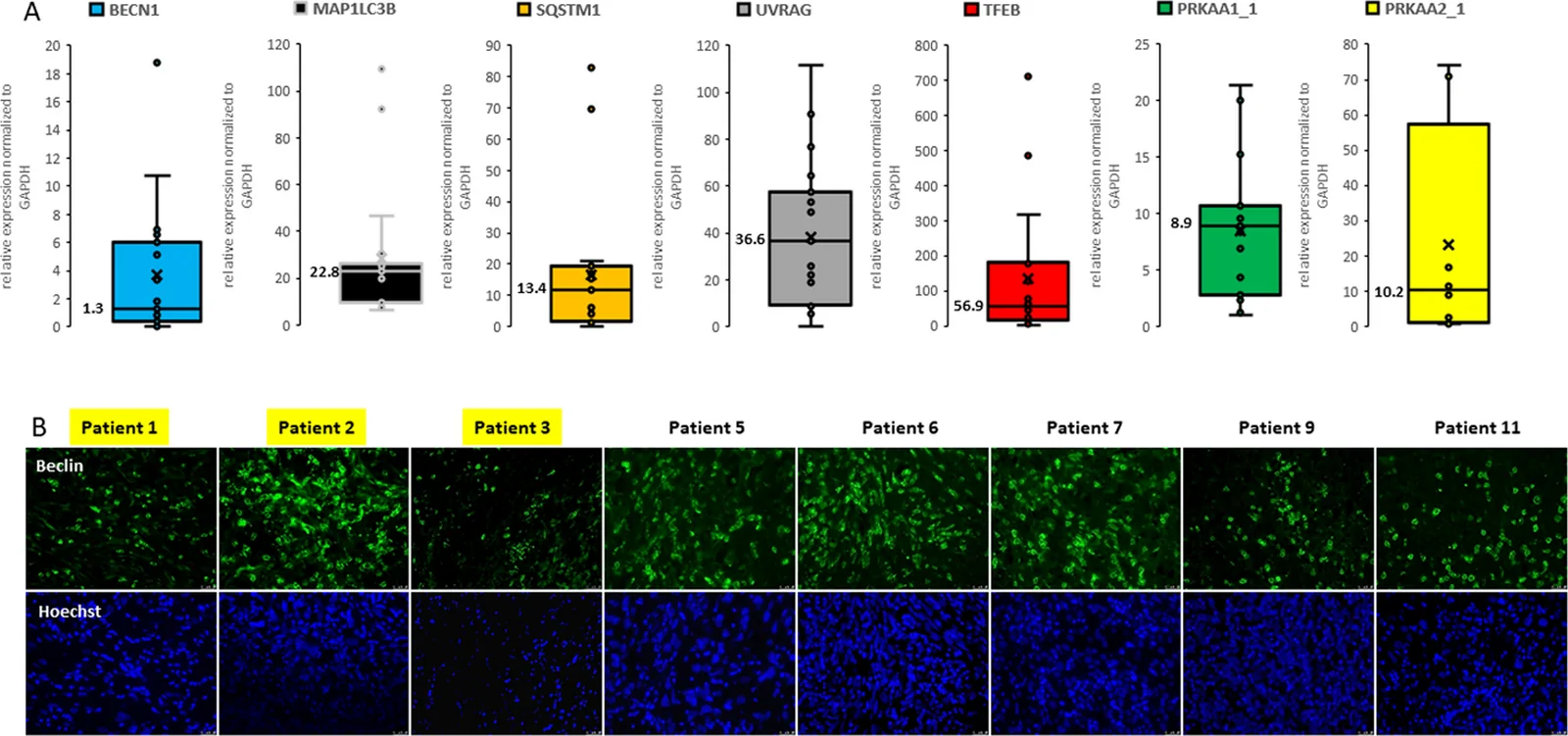
Detection of autophagy markers in anaplastic thyroid cancer tissue. **A** Box and whisker plots of *BECN1*, *MAP1LC3B*, *SQSTM1*, *UVRAG*, *TFEB*, *PRKAA1_1* and *PRKAA2_1* transcript levels. RNA was isolated from human ATC tissue. The tumor tissue expression was normalized to that of human follicular epithelial thyroid cells. The expression of the autophagy transcripts was normalized to that of GAPDH. The log10 means ± SEMs of triplicate samples are shown. 2-way ANOVA and Dunnett’s post hoc test for paired samples. *P* < 0.05 was considered to indicate statistical significance. **B** Immunofluorescence detection of Beclin in anaplastic thyroid cancer tissue. Tissue slices (10 µm) were deparaffinized and stained with a primary antibody against Beclin. The secondary antibody conjugated with Alexa Fluor 488 was used to identify the target protein by visualizing the green fluorescent spots. Nuclei were stained with Hoechst 33,342 (blue). The magnification is 20 ×

In addition to the several autophagy genes that transcribe proteins involved in the autophagosome synthesis process and catabolic activity, this study focused on the detection of the *BECN1*, *MAP1LC3B*, *UVRAG* and *SQSTM1* genes, which are responsible for autophagosome vesicle nucleation, maturation and elongation, as previously described [4, 29]. Additionally, the study focused on the detection of the transcription factor EB (*TFEB*), which is responsible for the transcription of autophagy genes, and the detection of *PRKAA1_1* and *PRKAA2_1*, the two genes responsible for the transcription of the two subunits of the kinase AMPKα (cyclic adenosine monophosphate kinase), which is a key element in linking metabolic signaling to autophagosome formation [4, 22]. As shown in Fig. 1A, the *BECN1* transcript was detectable in all 19 patient samples included in the study, and its expression was more stable than that in human follicular thyroid cells. Furthermore, the expression levels of *MAP1LC3B*, *UVRAG* and *SQSTM1* were significantly upregulated (median value 22.8-fold,13.4-fold; 36.6-fold) (Supplementary Table 1). Nonetheless, the *TFEB* transcript was also significantly overexpressed (median value 56.9-fold) (Supplementary Table 1). The transcripts for AMPKα, *PRKAA1_1* and *PRKAA2_1* were both overexpressed (8.9- and 10.2-fold median values) (Supplementary Table 1). However, *PRKAA2_1* was detectable in only 9 tumor tissue samples. The protein Beclin1 was detected by immunofluorescence in 10 patient samples. Figure 1B (Supplementary Fig. 3) highlights the overall expression of Beclin1 in eight out of the 10 patients affected by ATC. Lower magnification micrographs of all 10 patients are included in Suppl. Fig. 1.

### Photon irradiation induces the overexpression of autophagy-related gene transcripts

**Fig. 2:**
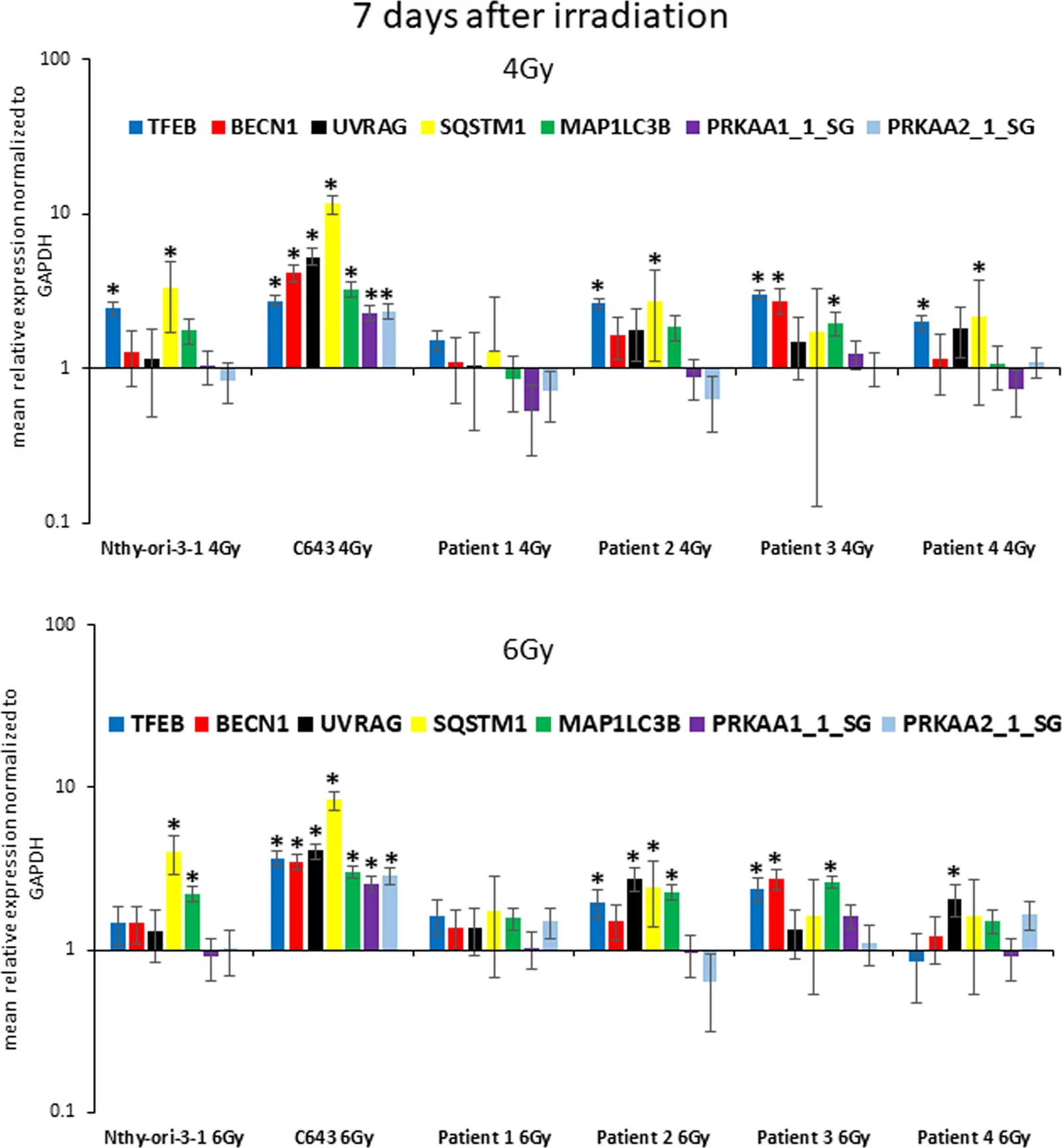
Expression of autophagy transcripts in irradiated anaplastic thyroid cancer cells. Transcript levels of *TFEB*, *BECN1*, *UVRAG*, *SQSTM1*, *MAP1LC3B*, *PRKAA1_1* and *PRKAA2_1* in Nthy-ori-3–1, C643, Patient 1, Patient 2, Patient 3 and Patient 4 cells 7 days after photon irradiation with 4 or 6 Gy. The irradiated cells were normalized to the untreated cells. GAPDH was detected as a housekeeping gene. 2-way ANOVA and Dunnett’s post hoc test for paired samples. *P* < 0.05 was considered to indicate statistical significance (*). *p < 0.05 was regarded as significant for untreated vs. irradiated cells. The log10 means ± SEMs of triplicate samples are shown

C643 cells, four primary tumor-derived cell lines (Patients 1–4) and human follicular epithelial thyroid cells (Nthy-ori-3–1) were photon irradiated with a single dosage of 4 or 6 Gy. Seven days after exposure, the expression of the *TFEB*, *BECN1*, *UVRAG*, *SQSTM1*, *MAP1LC3B*, *PRKAA1_1* and *PRKAA2_1* genes was detected in all the cells (Fig. 2). Exposure to 4 Gy caused significant overexpression of *TFEB* and *SQSTM1* in Nthy-ori-3–1, C643, Patient 2 and Patient 4 cells (Fig. 2) (Supplementary Table 2). Additionally, C643 cells were characterized by the significant overexpression of all the autophagy-related transcripts (Fig. 2) (Supplementary Table 2). However, Patient 1 exhibited stable expression of all the targets, and Patient 3 exhibited significant overexpression of only the *TFEB*, *BECN1* and *MAP1LC3B* transcripts. Exposure to 6 Gy, similar to 4 Gy, caused significant overexpression of autophagy genes in all the cells (Fig. 2) (Supplementary Table 2). Notably, C643 cells were, once again, the most sensitive in terms of overexpressing the autophagy transcripts. Patient 1 cells once again exhibited stable expression of the autophagy transcripts after exposure to 6 Gy (Fig. 2) (Supplementary Table 2). The autophagy transcripts have been detected one day after exposure to 4 and 6 Gy. No significant changes of the transcripts level have been observed in irradiated cells compared to untreated cells (Supplementary Fig. 2).

### Photon irradiation affects the protein level of autophagy players

**Fig. 3:**
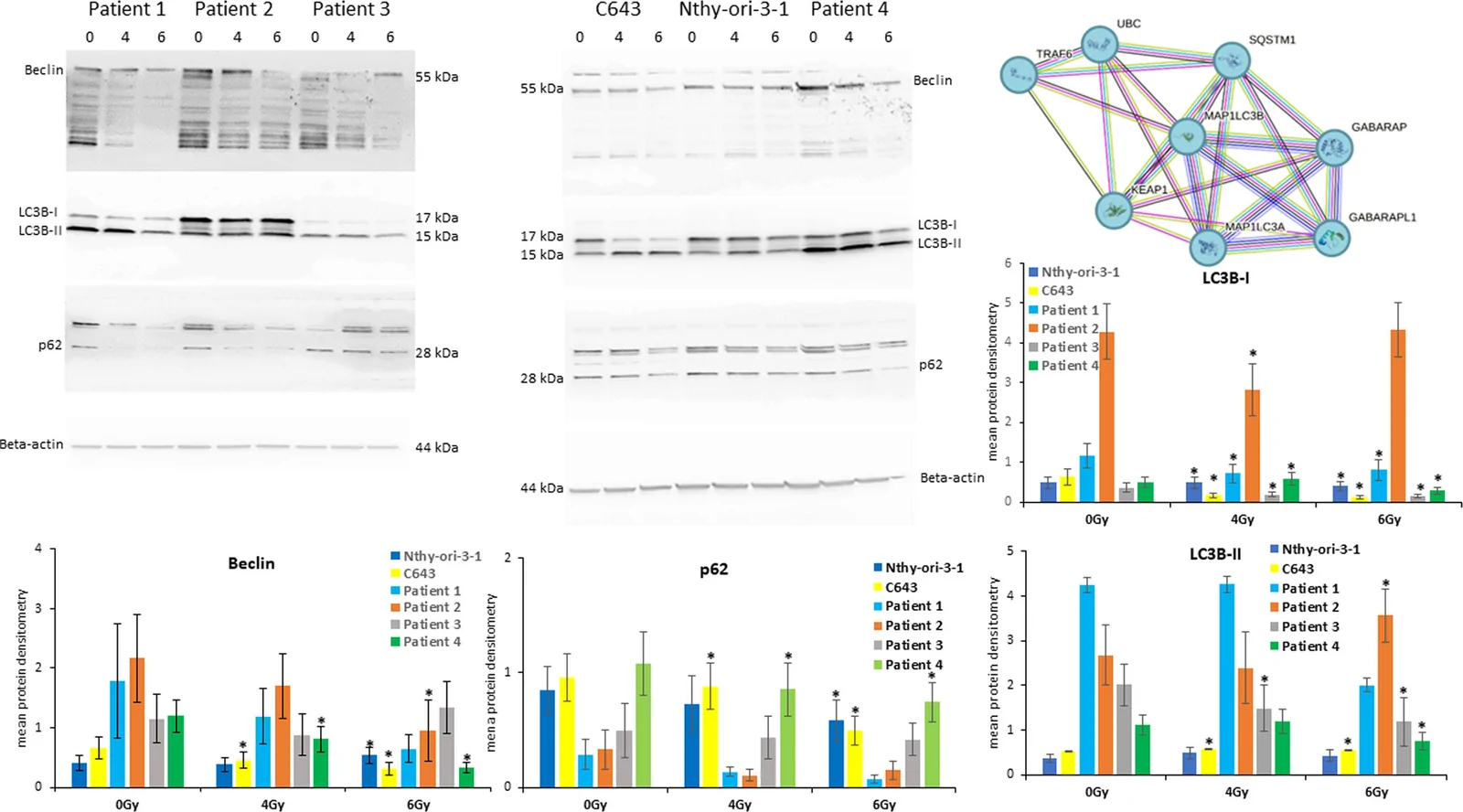
Detection of autophagy proteins in photon-irradiated ATCs. Western blot membranes (upper) and densitometry (lower) of Beclin, LC3B-I, LC3B-II and p62 proteins from Nthy-ori-3–1, C643, Patient 1, Patient 2, Patient 3 and Patient 4 cells 7 days after photon irradiation at 4 and 6 Gy. Densitometry results were normalized to the beta-actin content. The mean densitometry values are presented in triplicate ± SEM. 2-way ANOVA and Dunnett’s post hoc test for paired samples. *P* < 0.05 was considered to indicate statistical significance (*). *p < 0.05 was considered to indicate statistical significance: untreated vs 4 or 6 Gy-irradiated cells. The map of the interactions between autophagy proteins was constructed with STRING v12.0

Based on the current findings showing the ability of irradiation to modulate autophagy at the transcriptional level, further experiments have focused on detecting the protein levels of autophagy factors in irradiated anaplastic thyroid cancer cells. As shown in Fig. 3, exposure to 4 Gy caused the downregulation of Beclin, p62 and LC3B-I in all the anaplastic thyroid cancer cells (Supplementary Table 3). However, the protein level of LC3B-II was stable or slightly decreased (patient 3 cells). Nthy-ori-3–1 cells were the only cells that stably expressed all four proteins (Fig. 3). Irradiation with 6 Gy also downregulated Beclin, p62, LC3B-I and LC3B-II in all cancer cells (Fig. 3). Only Patient 2 cells were characterized by a stable protein level of LC3B-I and a significant increase in LC3B-II (Fig. 3). The protein levels of the autophagy proteins were not altered after irradiation with 4 Gy in human follicular epithelial thyroid cells. Only 6 Gy irradiation caused a significant increase in Beclin and a significant decrease in p62 in the non-tumor cells (Fig. 3) (Supplementary Table 3).

### Modulation of AMPKa after irradiation of ATC cells

**Fig. 4:**
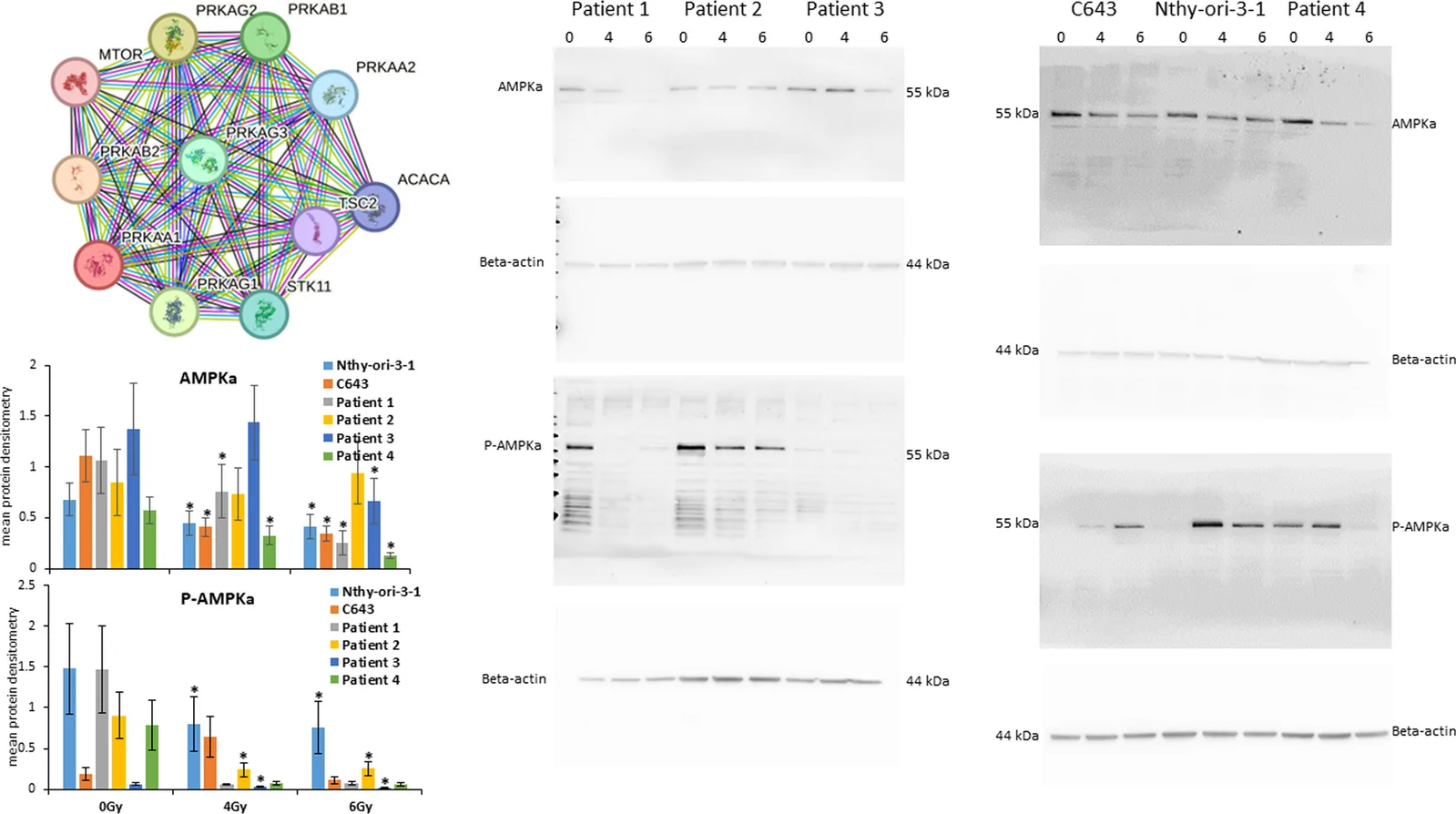
Detection of the AMPKα protein in photon-irradiated ATCs. Western blot membranes (upper) and densitometry (lower) of AMPKa and its active phosphorylated form P-AMPKa in Nthy-ori-3–1, C643, Patient 1, Patient 2, Patient 3 and Patient 4 cells 7 days after photon irradiation with 4 or 6 Gy. Densitometry results were normalized to the beta-actin content. The mean densitometry values are presented in triplicate ± SEM. 2-way ANOVA and Dunnett’s post hoc test for paired samples. *P* < 0.05 was considered to indicate statistical significance (*). *p < 0.05 was considered to indicate statistical significance: untreated vs 4 or 6 Gy-irradiated cells. A map of the PRKAA interaction was constructed with STRING v12.0

cAMP kinase alpha is responsible for the phosphorylation of Unc-51 like autophagy activating kinase 1 (ULK1) and for signaling related to autophagosome formation. Recently, autophagy was shown to act independently of AMPKα in cancer [22, 29], and this kinase has been shown to inhibit autophagy [24]. Nonetheless, alterations in the expression of the downregulated gene *PRKAA1* have recently been correlated with gastric and colorectal cancer risk and progression [3, 14, 23, 35]. Here, it was observed that the ATC cells and the Nthy-ori-3–1 cells exposed to 4 and 6 Gy were characterized by the downregulation of AMPKα (Fig. 4) (Supplementary Table 3). In particular, all the cells isolated 7 days after irradiation exhibited significant downregulation of the protein level of AMPKα, with the exception of Patient 4 cells, which exhibited stable expression (Fig. 4) (Supplementary Table 3). Furthermore, the active phosphorylated form of AMPKα (P-AMPKα) was downregulated by irradiation at both 4 and 6 Gy in all the cells Fig. 4) (Supplementary Table 3). Thus, irradiation was able to inhibit the activity of AMPKα not only by suppressing the total protein concentration but also by downregulating its active form.

### Live monitoring of the autophagy process in photon-irradiated ATCs

**Fig. 5:**
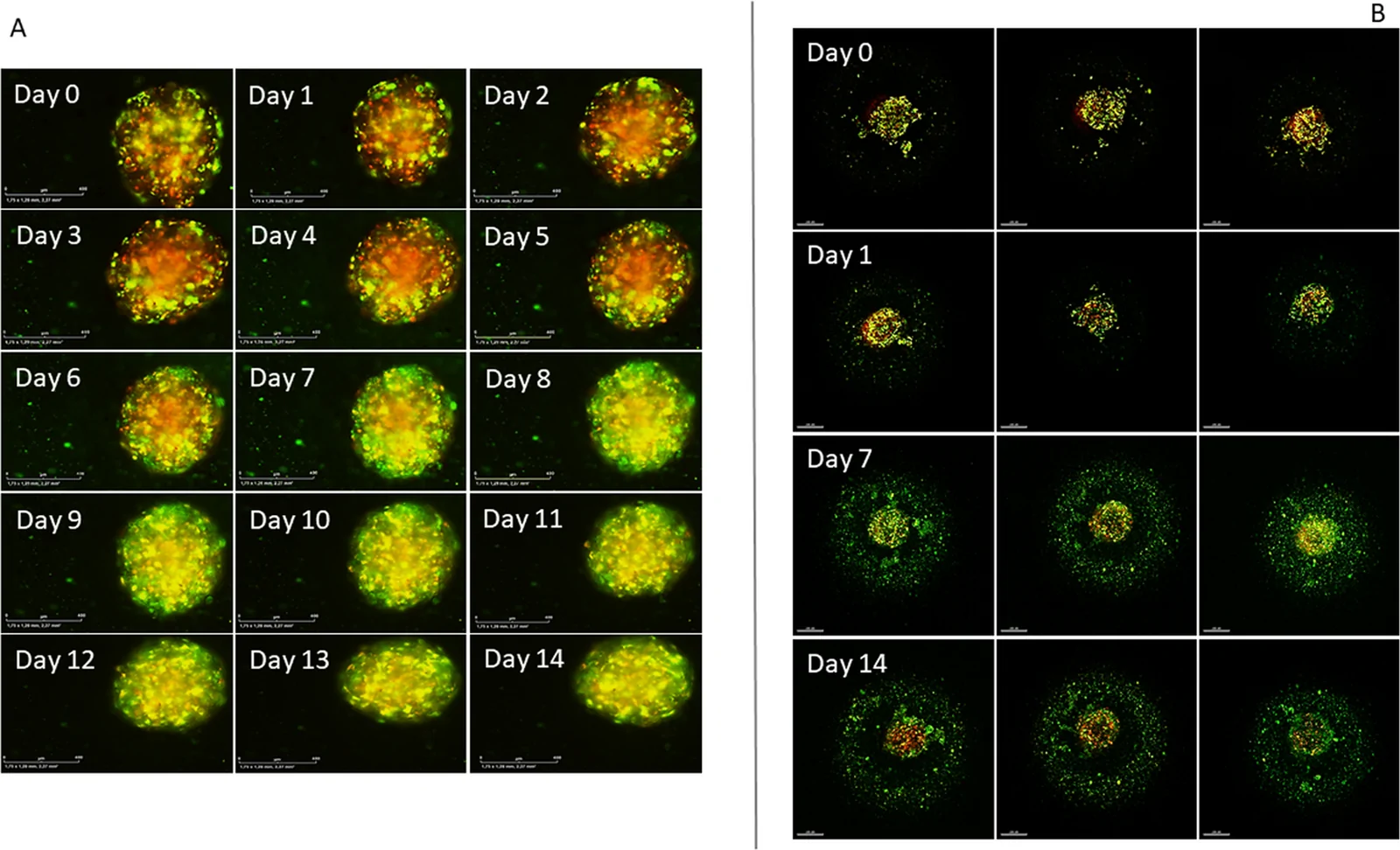
Fluorescence-based monitoring of autophagy in photon-irradiated ATCs. Stably transfected C643 cells were seeded in a Corning spheroid microplate. The cells were irradiated with 6 Gy. The green/red fluorescence was monitored for 28 days. The micrographs show fluorescence up to 14 days. (Left) Incucyte micrographs; the scale bar represents 400 µm. The magnification is 10 ×. (Right) Confocal microscopy micrographs; scale bar = 300 µm; magnification = 5x

C643 cells stably transfected with MAP1LC3B-GFP-RFP were seeded in a round bottom low-attachment plate for 4 days prior to exposure to 6 Gy photon irradiation. Immediately after exposure to 6 Gy photon irradiation, the C643 spheroid fluorescence and morphology were continuously tracked with IncuCyte for 28 days (Fig. 5A). Double-labeled LC3B allows the detection of autophagic maturation and terminal degradation activity after fusion with the lysosome. The terminal fusion of autophagosome vesicles and lysosomes causes the degradation of LC3B and acid-sensitive GFP*.* Instead, acid-stable RFP retains its fluorescence. As shown in Fig. 5 A, C643 cells exhibited basal autophagy at the time of exposure to 6 Gy photon irradiation, as indicated by consistent green and red fluorescence. The fluorescence intensity increased every day, and both fluorescence colors merged, as highlighted by the yellow‒brownish color after day 6 of exposure, resulting from the combination of green and red fluorescence. After 7 days of exposure to 6 Gy, the spheroid morphology started to dismantle, losing its three-dimensional structure and becoming untightened, while the green/red fluorescence remained stable (Suppl. Video). Similar results were observed via confocal microscopy (Fig. 5B). In particular, these micrographs provided more detailed evidence of the dismantling of the spheroid morphology and the increase in fluorescence. This result supports previous findings highlighting the efficacy of irradiation in promoting autophagy, especially 7 days after exposure.

## Discussion

Anaplastic thyroid cancer is characterized by an extremely poor prognosis. Despite recent discoveries about the efficacy of personalized therapy based on mutation screening, patients affected by ATC still need therapy to significantly improve their survival rate. Combined therapy with lenvatinib, a tyrosine kinase inhibitor; pembrolizumab, a monoclonal antibody against PD-1; and the combination of dabrafenib, a BRAF inhibitor; and trametinib, a MAPK inhibitor, has shown promising effects on these patients [2]. Unfortunately, prolonged treatment has led to relapse of the malignancy, thus affecting patient progression-free survival [36]. For this reason, a second-line/adjuvant therapy is strongly needed to overcome the loss of efficacy of first-line therapy. The efficacy of radiotherapy in treating ATC has been highlighted previously [15, 30, 37]. In particular, our previous study demonstrated that photon therapy is able to block the proliferation of ATC cells, thus preventing colony formation. The study could exclude the possibility of apoptosis as a cell death mechanism after irradiation. In fact, radiation can increase the protein level of PD-L1. Its inhibition mediated by the administration of atezolizumab exacerbates the inhibitory effect of radiotherapy. Thus, other cellular processes could be involved in the cellular decay prompted by photon irradiation. The current study focused on the ability of photon irradiation to promote autophagy as an alternative cell death mechanism. Autophagic cell death has been previously shown to be induced in anaplastic thyroid cancer cells by the administration of different compounds, thus revealing that this catabolic process is a promising therapeutic target for ATC [29]. The combination of tyrosine kinase or pan-deacetylase inhibitors with PD-L1 blockers, as well as berberine or even piperlongumine and capsaicin, has shown the ability to lead to autophagic cell death [18, 27, 29, 34]. Here, it was shown for the first time that photon irradiation promoted autophagy in ATC cells.

Importantly, the radiation doses applied in this study (4 and 6 Gy per fraction) should be interpreted in the context of both preclinical modeling and current clinical strategies for ATC. Although standard fractionated radiotherapy is commonly delivered at 2 Gy per fraction, ATC is characterized by marked radioresistance and aggressive local progression, prompting the clinical exploration of dose escalation, hypofractionated schedules, and stereotactic body radiotherapy approaches. In this setting, single or few fractions delivering higher biologically effective doses are increasingly considered to improve local control, as reflected in current clinical recommendations (AWMF Guideline Schilddrüsenkarzinom 2025), [1, 25]. Modeling 4–6 Gy in vitro therefore reflects radiation intensities encountered in modern ATC treatment concepts and enables the investigation of biologically relevant radiation responses under clinically meaningful conditions.

In particular, after seven days, 4 and 6 Gy irradiation caused the overexpression of the transcripts of the most related autophagy genes, *TFEB, BECN1, UVRAG, SQSTM1 MAP1LC3B, PRKAA1_1* and *PRKAA2_1*. Additionally, the protein levels of the autophagy players Beclin, LC3B-I, LC3B-II and p62 were significantly downregulated seven days after photon irradiation. The protein levels of AMPKα and its active phosphorylated form were also downregulated in irradiated cells. Thus, excluding definitively, its role in autophagy modulation. Similar effects of AMPKα have already been shown in pancreatic neuroendocrine neoplasia, where AKT exerts an inhibitory effect on AMPKα [22]. However, these findings do not imply a negative modulation of autophagy that could be promoted at the transcriptional level by the cAMP responsive element or by the transcription factor TFEB [22]. Moreover, after irradiation, autophagy continued to occur, and the process was further promoted, as shown by the increase in double fluorescence. This leads to a progressive dismantle of the ultrastructure of C643 cell-derived spheroids and ultimately cell decay. Anaplastic thyroid cancer tissue resected from patients showed significant overexpression of the autophagy genes *MAP1LC3B, SQSTM1, UVRAG, TFEB, PRKAA1_1* and *PRKAA2_1* as well as the protein Beclin1. Thus, ATC is characterized by stable active autophagy that can be promoted to induce cell death. This option has been previously shown for several solid cancers [5, 21, 22]. The expression of autophagy genes has been normalized to normal follicular immortalized thyrocites, which represents the great limitation but the best option considering that would not be possible to isolate thyroid tissue from healthy patients. However, the para tumour tissue of the patients affected by ATC is commonly high heterogeneous, as already reported [16], thus affecting the results reported in this study. Furthermore, autophagic cell death can promote cell death when prompted by small drugs and immune checkpoint inhibitors, not only in anaplastic thyroid cancer [29] but also in other solid malignancies [6, 33]. Additionally, autophagy is likely involved in anaplastic lymphoma kinase (ALK)-associated cancers [8]. Future work should explore whether autophagy modulation synergizes with ALK inhibitors in ATC.

## Conclusions

This study showed, for the first time, the ability of photon irradiation to sensitize anaplastic thyroid cancer cells to autophagy. Thus, cell death occurs. It is not known whether irradiation-mediated autophagy can modulate PD-L1 in ATCs, as previously shown [9]. Future work should explore crosstalk between irradiation-induced autophagy and immune checkpoint pathways like PD-L1.

## Supplementary Information

Supplementary Material 1.

Supplementary Material 2.

Supplementary Material 3.

Supplementary Material 4.

Supplementary Material 5.

## Data Availability

The datasets used and/or analyzed during the current study are available from the corresponding author upon reasonable request.

## Abbreviations

ATC: Anaplastic Thyroid Cancer
GFP: Green fluorescent protein
RFP: Red fluorescent protein
LC3B: Light Chain 3B
AMPKα: 5'-AMP-activated protein kinase alpha
IMRT: Intensity-modulated radiotherapy
DSBs: Double Strand Breaks
ICI: Immune checkpoint inhibitor
mKI: Multikinase Inhibitor
RPMI: Roswell Park Memorial Institute
FFPE: Formalin Fixed Paraffin Embedded
PDTT: Patient-Derived Tumor Tissue
PBS: Phosphate-buffered saline
FBS: Fetal bovine serum
BSA: Bovine Serum Albumin
UVRAG: UV Radiation Resistance-Associated Gene
SQSTM1: Sequestosome 1
TFEB: Transcription Factor EB
PRKAA1_1: 5'-AMP-activated protein kinase catalytic subunit alpha-1
PRKAA2_1: 5'-AMP-activated protein kinase catalytic subunit alpha-2
GAPDH: Glyceraldehyde 3-Phosphate Dehydrogenase
MAP1LC3B: Microtubule-associated protein 1A/1B light chain 3B
BECN1: Beclin1
PD-1: Programmed cell death protein 1
PD-L1: Programmed death-ligand 1
