# Supplementary material for: Photon irradiation prompts autophagy in anaplastic thyroid cancer
Source: BMC Cancer. 2026 Aug 25;26:1021. doi: 10.1186/s12885-026-16762-0 (PMC13508399; doi:10.1186/s12885-026-16762-0)
Supplement: Supplementary file 1 [file 12885_2026_16762_MOESM1_ESM.docx]

|  | BECN1 | TFEB | SQSTM1 | MAP1LC3B | UVRAG | PRKAA1_1 | PRKAA2_1 |
| --- | --- | --- | --- | --- | --- | --- | --- |
| Control vs. 26 | 0.0087 | 0.0014 | 0.0913 | 0.002 | <0.0001 | 0,0002 | 0,0001 |
| Control vs. 27 | <0.0001 | <0.0001 | <0.0001 | <0.0001 | <0.0001 | <0.0001 |  |
| Control vs. 28 | <0.0001 | <0.0001 | <0.0001 | <0.0001 | <0.0001 | <0.0001 |  |
| Control vs. 29 | 0.0032 | <0.0001 | <0.0001 | <0.0001 | <0.0001 | <0.0001 |  |
| Control vs. 30 | 0.0231 | <0.0001 | 0.0014 | <0.0001 | <0.0001 | <0.0001 | <0.0001 |
| Control vs. 31 | 0.0011 | <0.0001 | <0.0001 | <0.0001 | <0.0001 | <0.0001 | 0,0006 |
| Control vs. 33 | 0.0825 | 0.0018 | 0.0411 | <0.0001 | <0.0001 | <0.0001 | 0,0011 |
| Control vs. 34 | 0.0002 | <0.0001 | <0.0001 | <0.0001 | <0.0001 | <0.0001 |  |
| Control vs. 35 | 0.0009 | <0.0001 | <0.0001 | <0.0001 | <0.0001 | <0.0001 |  |
| Control vs. 36 | 0.0003 | <0.0001 | <0.0001 | <0.0001 | <0.0001 | <0.0001 |  |
| Control vs. 38 | <0.0001 | <0.0001 | <0.0001 | <0.0001 | <0.0001 | <0.0001 | <0.0001 |
| Control vs. 39 | 0.0001 | 0.0279 | 0.0007 | <0.0001 | 0.0006 | 0,9998 | <0.0001 |
| Control vs. 40 | 0.0003 | <0.0001 | 0.2157 | <0.0001 | <0.0001 | <0.0001 |  |
| Control vs. 41 | <0.0001 | <0.0001 | <0.0001 | <0.0001 | <0.0001 | <0.0001 |  |
| Control vs. 42 | 0.007 | <0.0001 | <0.0001 | 0.057 | <0.0001 | <0.0001 | <0.0001 |
| Control vs. 44 | 0.7515 | 0.0014 | 0.0002 | <0.0001 | <0.0001 | <0.0001 | <0.0001 |
| Control vs. 45 | 0.0059 | <0.0001 | <0.0001 | <0.0001 | <0.0001 | <0.0001 | <0.0001 |
| Control vs. 50 | 0.8115 | <0.0001 | 0.0211 | 0.0311 | 0.0001 | <0.0001 | 0,0006 |

Supplementary Table 1

Statistical analysis of PCR results of ATC patients. The expression of the transcripts has been normalized versus untreated Nthy-ori-3-1 cells. 2-way ANOVA with Dunnett`s multiple comparison test has been performed for paired samples. *p < 0.05 was regarded as significant.

Supplementary Table 2

|  |  | C643 | N-THY-ori 3 | Patient 1 | Patient 2 | Patient 3 | Patient 4 |
| --- | --- | --- | --- | --- | --- | --- | --- |
| TEFB | 0 vs. 4 | 0,0018 | <0.0001 | 0,0023 | 0,0004 | 0,0008 | <0.0001 |
|  | 0 vs. 6 | 0,0028 | 0,0007 | 0,0258 | 0,0001 | 0,0373 | <0.0001 |
|  |  |  |  |  |  |  |  |
| BECN1 | 0 vs. 4 | 0,0006 | <0.0001 | 0,0082 | 0,0003 | 0,0001 | 0,6986 |
|  | 0 vs. 6 | 0,0003 | 0,0041 | <0.0001 | 0,0066 | <0.0001 | 0,0223 |
|  |  |  |  |  |  |  |  |
| UVRAG | 0 vs. 4 | 0,0267 | 0,0763 | 0,0822 | 0,9214 | 0,0029 | 0,0073 |
|  | 0 vs. 6 | 0,0441 | 0,6431 | 0,1078 | 0,2868 | 0,0019 | 0,0003 |
|  |  |  |  |  |  |  |  |
| SQSTM1 | 0 vs. 4 | <0.0001 | 0,0008 | 0,0034 | 0,0008 | 0,0006 | <0.0001 |
|  | 0 vs. 6 | <0.0001 | <0.0001 | 0,0023 | <0.0001 | 0,0006 | <0.0001 |
|  |  |  |  |  |  |  |  |
| MAP1LC3B | 0 vs. 4 | 0,0047 | <0.0001 | 0,0584 | 0,0659 | 0,0082 | 0,0009 |
|  | 0 vs. 6 | 0,0887 | 0,0309 | 0,0141 | 0,0002 | 0,006 | <0.0001 |
|  |  |  |  |  |  |  |  |
| PRKAA1_1_SG | 0 vs. 4 | 0,0002 | <0.0001 | <0.0001 | <0.0001 | 0,0014 | 0,0003 |
|  | 0 vs. 6 | 0,0222 | 0,0002 | 0,0014 | <0.0001 | 0,0247 | 0,1285 |
|  |  |  |  |  |  |  |  |
| PRKAA2_1_SG | 0 vs. 4 | 0,0008 | <0.0001 | 0,0008 | <0.0001 | <0.0001 | 0,1291 |
|  | 0 vs. 6 | 0,9039 | <0.0001 | 0,0159 | <0.0001 | <0.0001 | 0,0003 |

Statistical analysis of PCR results of C643, Nthy-ori-3-1, Patient1, Patient 2, Patient 3 and Patient 4 cells 7 days after single photon irradiation with 4 and 6 Gy.. The expression of the transcripts has been normalized versus untreated cells. 2-way ANOVA with Dunnett`s multiple comparison test has been performed for paired samples. *p < 0.05 was regarded as significant.

Supplementary Table 3

|  |  | C643 | N-THY-ori 3 | Patient 4 | Patient 1 | Patient 2 | Patient 3 |
| --- | --- | --- | --- | --- | --- | --- | --- |
| Beclin | 0 vs. 4 | *0,0413 | 0,5665 | *0,065 | 0,4884 | 0,0913 | 0,3217 |
|  | 0 vs. 6 | *0,007 | *0,0163 | *0,0043 | 0,1622 | *0,0038 | 0,5436 |
|  |  |  |  |  |  |  |  |
| LC3B-I | 0 vs. 4 | *0,0296 | >0.9999 | 0,3069 | *0,0013 | *<0.0001 | *0,0292 |
|  | 0 vs. 6 | *0,0225 | *0,0212 | *0,0333 | *0,003 | 0,867 | *0,0184 |
|  |  |  |  |  |  |  |  |
| LC3B-II | 0 vs. 4 | *0,0294 | *0,0363 | 0,2438 | 0,9954 | 0,41 | *0,0313 |
|  | 0 vs. 6 | *0,1343 | 0,1892 | *0,0023 | *0,0003 | *0,029 | *0,0064 |
|  |  |  |  |  |  |  |  |
| p62 | 0 vs. 4 | 0,6041 | 0,2573 | 0,0846 | 0,1991 | 0,1001 | 0,6242 |
|  | 0 vs. 6 | *0,0079 | *0,0303 | *0,024 | 0,0877 | 0,1704 | 0,4898 |
|  |  |  |  |  |  |  |  |
| AMPK | 0 vs. 4 | *0,014 | *0,0163 | 0,0522 | 0,1877 | 0,378 | 0,8962 |
|  | 0 vs. 6 | *0,0102 | *0,0105 | *0,0074 | *0,0107 | 0,443 | *0,0194 |
|  |  |  |  |  |  |  |  |
| P-AMPK | 0 vs. 4 | 0,0756 | *0,0388 | 0,0641 | 0,0533 | *0,0248 | *0,0018 |
|  | 0 vs. 6 | 0,8459 | *0,0312 | 0,0619 | 0,0553 | *0,0266 | *0,0005 |

Statistical analysis of western blot densitometry results of C643, Nthy-ori-3-1, Patient 4, Patient 1, Patient 2 and Patient 3 cells 7 days after single photon irradiation with 4 and 6 Gy.. The expression of the proteins has been normalized versus untreated cells. 2-way ANOVA with Dunnett`s multiple comparison test has been performed for paired samples. *p < 0.05 was regarded as significant.
